# Late Onset Treatment Resistant Catatonia : A Diagnostic Challenge at the Psychiatry-Neurology Interface

**DOI:** 10.1192/bjo.2026.11773

**Published:** 2026-06-30

**Authors:** Rishabh Chormalle, Toby Greenall

**Affiliations:** Lincolnshire Partnership Foundation Trust, Lincoln, United Kingdom

## Abstract

**Aims:**

Late-onset treatment resistant psychosis with catatonia poses a significant diagnostic challenge, particularly where neurological and psychiatric features overlap.

Anti-n-Methyl-D-aspartate receptor {NMDAR} antibody-associated syndromes may present predominantly with psychiatric symptoms and delayed or fluctuating seropositivity further complicates diagnosis.

**Methods:**

We describe a patient in early 60s with no prior psychiatry history who developed a severe, relapsing neuropsychiatric illness a few years ago. Patient presented initially with depressive symptoms, health anxiety, weight loss and insomnia, followed by psychosis characterised by auditory hallucinations, nihilistic and homicidal delusions, behavioural disinhibition, and prominent catatonic features including mutism, psychomotor retardation, posturing and reduced responsiveness.

Over several years, patient underwent multiple prolonged hospital admissions under the Mental Health Act. The illness proved highly treatment resistant with limited or transient response to multiple antidepressants, antipsychotics, mood stabilisers, benzodiazepines and several courses of electroconvulsive therapy.

Lorazepam resulted in partially, short lived improvement in catatonic symptoms. Clozapine was discontinued as patient developed constipation. Between episodes, only brief periods of partial recovery were observed, with no sustained return to baseline functioning.

Extensive medical and neurological investigations were undertaken, including repeated MRI and CT brain imaging, EEG, Lumbar puncture, PET and DAT scan, and comprehensive autoimmune and paraneoplastic screening. Initial antibody testing for autoimmune psychosis was negative, however, subsequent repeated testing showed serum NMDR antibodies. Cerebrospinal fluid antibodies were negative.

Trials of corticosteroids and dopaminergic therapy were undertaken amid evolving differential and working diagnoses, including limbic encephalitis, Parkinsonian syndromes, dementia with Lewy Bodies and functional neurological disorder.

**Results:**

This case highlights the complexity of late onset psychosis with catatonia and the diagnostic uncertainty surrounding fluctuating NMDR antibody positivity and other neurological symptomology. It underscores the need for close collaboration between Psychiatry and Neurology, cautious interpretation or results, reassessments and revisiting the differential and working diagnoses over time.

**Conclusion:**

Persistent, treatment resistant catatonia in adults should prompt ongoing consideration of autoimmune and neurological differentials, despite inconclusive initial investigations. This case illustrates the challenges of diagnosis and management at the interface of Psychiatry and Neurology.

